# The role of dermal fibroblasts in autoimmune skin diseases

**DOI:** 10.3389/fimmu.2024.1379490

**Published:** 2024-03-13

**Authors:** Zeqi Shi, Zhong Liu, Yujia Wei, Ri Zhang, Yunhua Deng, Dong Li

**Affiliations:** Department of Dermatology, Tongji Hospital, Tongji Medical College, Huazhong University of Science and Technology, Wuhan, China

**Keywords:** dermal fibroblast, single-cell RNA sequencing, heterogeneity, autoimmune skin disease, immunology

## Abstract

Fibroblasts are an important subset of mesenchymal cells in maintaining skin homeostasis and resisting harmful stimuli. Meanwhile, fibroblasts modulate immune cell function by secreting cytokines, thereby implicating their involvement in various dermatological conditions such as psoriasis, vitiligo, and atopic dermatitis. Recently, variations in the subtypes of fibroblasts and their expression profiles have been identified in these prevalent autoimmune skin diseases, implying that fibroblasts may exhibit distinct functionalities across different diseases. In this review, from the perspective of their fundamental functions and remarkable heterogeneity, we have comprehensively collected evidence on the role of fibroblasts and their distinct subpopulations in psoriasis, vitiligo, atopic dermatitis, and scleroderma. Importantly, these findings hold promise for guiding future research directions and identifying novel therapeutic targets for treating these diseases.

## 1 Introduction

The skin is a vital organ, the first barrier, protecting the host from external pathogens and harmful stimuli. It consists of diverse cell types, including keratinocytes, melanocytes, fibroblasts, and immune cells, which collaborate to maintain skin homeostasis (1). Skin diseases rank fourth in terms of incidence among all causes of disease, affecting approximately one-third of the global population. However, the burden of skin diseases, particularly autoimmune-related diseases, is frequently underestimated despite their conspicuous nature (2). Autoimmune skin diseases are characterized by an aberrant immune response within the cutaneous tissue, leading to compromised structural and functional integrity. This process involves diverse cells, including macrophages, T cells, B cells, keratinocytes, and fibroblasts (3).

Fibroblasts, a crucial subset of mesenchymal cells, exhibit diverse biological functions (4). Within the skin, fibroblasts are primarily localized in the dermis and play an indispensable role in maintaining the structural integrity of the skin, regulating immune responses, and participating in cutaneous damage repair (5). In recent years, accumulating evidence has revealed the intricate involvement of fibroblasts in the pathogenesis and progression of various autoimmune skin diseases in human and mouse models. For instance, dermal fibroblasts play a pivotal role as a cellular source for inflammatory cytokines and chemokines, promoting chronic tissue inflammation through leukocyte recruitment and exacerbating inflammatory injury (4). Recently, histological approaches and single-cell RNA sequencing (scRNA-seq) studies on human skin diseases have revealed fibroblast subsets with unexpected immuno-modulatory transcriptomes and immune cell changes, suggesting a potential role of these cells in the pathogenesis of inflammatory skin disorders (4, 6). Whether skin fibroblasts contribute to immune homeostasis in skin inflammatory disorders remains unknown. Understanding the role of fibroblasts in these diseases may lead to the development of new therapeutic strategies.

In this review, we provide a brief overview of the basic function of dermal fibroblasts in the skin, as well as their essential role in pathological conditions. We also present the current knowledge on the subpopulation of fibroblasts and their function in various autoimmune skin diseases (**Table 1**).

**Table 1:** Summary of vital genes of fibroblast in autoimmune skin diseases.

Disease	Vital Gene	Function of gene	References
Psoriasis	*SFRP2*	product pro-inflammatory cytokines	7
	*TNC*	induce psoriasiform skin inflammation	8
	*OVOL1*	aggravate psoriasis-like skin inflammation	9
Vitiligo	*CXCL9* *CXCL10*	recruit T cell	10, 11
	*CCL2* *CCL8*	promoted naïve T cell polarization into Th2 cells, and attract Th2 cells	12
Atopic Dermatitis	*COL6A51*	recruit T cell	13
	*COL18A11*	product type2 inflammation signal induced cytokines	13
Scleroderma	*Prx1*	product pro-inflammatory cytokines	14
	*LGR5*	coordinate the correct tissue organization and homeostasis	15
	*SFRP2*	differentiate into myofibroblasts	16

## 2 Dermal fibroblasts

### 2.1 Function of dermal fibroblasts

Fibroblasts perform a diverse array of functions within various tissues and organs, encompassing the secretion and remodeling of extracellular matrix (ECM), generation of mechanical force, regulation of tissue metabolism, as well as the secretion of signaling factors that modulate immune responses and maintain skin micro-environmental hemostasis (17–19).

**Figure 1:**
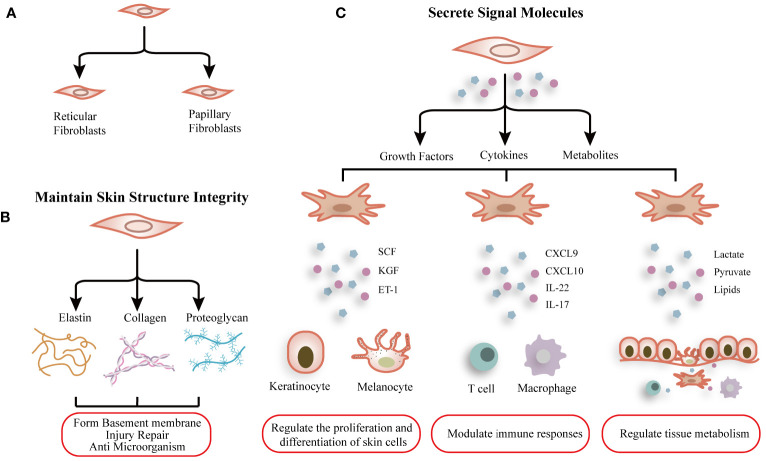
Schematic graph showing the function and heterogeneity of dermal fibroblasts. **(A)** Normal dermal fibroblasts are divided into two subgroups: Reticular fibroblasts and Papillary fibroblasts. **(B)** Fibroblasts synthesize extracellular matrix to maintain skin structure integrity. **(C)** Fibroblasts modulate the microenvironment of the dermis and the immune responses by secreting numerous cytokines, metabolites, and growth factors.

The dermis is the most crucial layer of skin in the skin and harbors fibroblasts as the predominant cell type. The primary function of fibroblasts is to synthesize and ECM (collagens, elastin, fibronectin, laminins, et al.) (**Figure 1B**), contributing to connective tissue formation that maintains skin structure integrity and function (20). Meanwhile, fibroblasts modulated the microenvironment of the dermis and the immune responses by secreting numerous cytokines, metabolites, and growth factors (21). The fibroblasts exhibit robust capabilities in proliferation, differentiation, and migration. In response to detrimental stimuli, they can differentiate into myofibroblasts and secrete ECM and cytokines in order to counteract external damage and restore skin function (**Figure 1C**). On the contrary, fibroblast dysfunction also contributes to skin diseases such as psoriasis and atopic dermatitis.

### 2.2 Heterogeneity and subtypes of fibroblasts

Fibroblasts are mesenchymal cells that exhibit remarkable plasticity and can be differentiated from a variety of cells in response to external stimuli (22). Over past decades, extensive research has demonstrated that fibroblasts exhibit high heterogeneity (23). However, in the early stages, the lack of advanced technological support presents a significant challenge for distinguishing subpopulations of fibroblasts. Recently, advancements in single-cell sequencing technology have identified numerous subtypes within fibroblasts.

Normal dermal fibroblasts are divided into two subgroups, papillary fibroblasts and reticular fibroblasts (**Figure 1A**), and the common markers are COL1A1, COL1A2 et al. Korosec found that fibroblasts characterized by fibroblast activation protein (FAP) positive and CD90 negative phenotype are enriched in the papillary dermis and express both PDPN and NTN1, display active proliferation, and are relatively resistant to adipogenic differentiation. On the other hand, FAP-CD90^+^ fibroblasts expressed high levels of ACTA2, MGP, PPARγ, and CD36 and possessed a higher adipogenic potential, contributing to features of reticular fibroblasts (24).

A single-cell RNA sequencing study showed that dermal fibroblasts can be grouped into two main subpopulations by SFRP2 and FMO1 (7). However, these two subtypes are not associated with the papillary and reticular layers. Philippeos et al. found that CD90, platelet-derived growth factor receptor (PDGFR) were the hallmarks of fibroblasts, and human dermal fibroblasts have been divided into four subgroups based on their location in the dermis (25).

## 3 The role of dermal fibroblasts in autoimmune skin diseases

### 3.1 Dermal fibroblasts in psoriasis

Psoriasis is a worldwide autoimmune skin disease, caused by immune system malfunction, resulting in red, scaly patches and excessive skin exfoliation due to increased keratinocyte proliferation (26, 27). While the involvement of keratinocytes and IL-17-producing helper T (Th17) cells in psoriasis has been extensively described (28, 29), limited attention has been devoted to other cell types involved in psoriasis. Recent studies have focused on the crucial role of fibroblasts in the development of psoriatic lesions.

Fibroblasts isolated from psoriatic skin possess a distinct gene expression profile. Kim et al. revealed that ITGA4 expression was significantly upregulated in psoriatic fibroblasts (30). Kyoto Encyclopedia of Genes and Genomes (KEGG) analysis revealed that ECM-receptor interaction was the most enriched pathway in psoriatic fibroblasts, highlighting their crucial role in producing ECM molecules. Meanwhile, extra domain A fibronectin (EDA FN), a sign of dormant chronic inflammation, also had a significant upregulation in psoriatic fibroblasts (30, 31). In psoriatic mice with OVOL1 or Vsir deficiency, fibroblasts emerged as one of the major cell types involved (9, 32). OVOL1 deficiency led to the upregulation of inflammation-associated genes such as Saa3 and monocyte chemoattractant Ccl2 in fibroblasts, aggravating psoriasis-like skin inflammation (9, 33). Fibroblasts in IMQ-treated Ovol1-deficient skin lost the capacity to inhibit IL-1 signaling, resulting in hyperactivated inflammation (9). Similarly, Ptx3, Hsbp1, Cebpb, and Tnfrsf12a were elevated in fibroblasts from Vsir-deficient psoriatic mouse skin, whereas S100a9 was downregulated. These genes were enriched for macrophage-associated genes, inflammatory response, and wound healing (32). Cai et al. have revealed that the expression of adipogenic, pro-inflammatory, and chemotactic genes were significantly upregulated in IMQ-induced fibroblasts and a notable skewing towards profiles adapted to inflammation (8). Results from scRNA-seq in IMQ-induced psoriatic skin confirmed the essential role of fibroblasts in psoriasis pathogenesis.

However, prior studies on the role of fibroblasts have mostly been limited to the ability of fibroblasts to induce keratinocyte proliferation and regulate immune responses (34). Following that, subsequent research has provided detailed insights into the role of different subsets of fibroblasts in psoriasis pathogenesis, identifying the fibroblast subpopulations involved in psoriasis.

SFRP2^+^ fibroblasts have been identified as essential cells in psoriasis pathogenesis, which impacts other spatially proximate cell types by producing chemokines (35). SFRP2^+^ fibroblasts were divided into two groups: one producing extracellular matrix components and the other with a pro-inflammatory phenotype. This study also demonstrated that SFRP2^+^ fibroblasts are a source of proteases such as cathepsin S, which activates IL-36γ (36). Meanwhile, expression of pro-inflammatory cytokines in fibroblast was predominantly localized to the tips of the dermal papillae, an area within psoriatic skin known for harboring inflammatory cells and being in close proximity to the overlying epidermis (37). In murine skin, a population of inflammatory fibroblasts that shared similarities with SFRP2^+^ fibroblasts and expressed genes associated with psoriasis-related inflammation was identified (38). Interestingly, SFRP2^+^ inflammatory fibroblasts in psoriasis and COL6A5^+^ COL18A1^+^ fibroblasts in atopic dermatitis express CCL19, which can recruit CCR7^+^ LAMP3^+^ type 2 conventional dendritic cell (cDC2) (13). However, only psoriatic fibroblasts expressed CXCL12 and CXCL1, which contributed to the recruitment of CXCR4^+^ Tc17 cells and neutrophils (35). Cai et al. reported a subpopulation of papillary fibroblasts induced by inflammation that secrete the extracellular matrix protein tenascin-C (TNC), facilitating psoriasiform skin inflammation. Specific ablation of TNC in fibroblasts reduces hyperinnervation and skin inflammation in male psoriasis mice (8).

Other omics studies have also revealed the important role of fibroblasts in psoriasis. Gegotek et al. investigated alterations in the proteomic profile of dermal fibroblasts within psoriasis lesions. The psoriatic fibroblasts exhibited upregulation of proinflammatory and antioxidant proteins, signal transduction molecules, and proteolytic enzymes. Conversely, downregulated proteins in psoriatic fibroblasts primarily encompassed those involved in transcription or translation processes, glycolysis/ATP synthesis, and structural support (39). The findings suggest that alterations in oxidative stress and protein expression within fibroblasts, along with their regulatory role in immune response, may contribute to the pathogenesis of psoriasis. By using the lipidomic method, Łuczaj et al. have assessed the adaptation of the ceramide profile of fibroblasts from psoriasis vulgaris patients. The research showed significant increases in the three ceramide classes (CER[AS], CER[ADS], and CER[EOS]), which were expressed at higher levels in psoriasis patients. The most noteworthy change in the fibroblasts was increased CER[EOS], which included ester-linked fatty acids (40).

### 3.2 Dermal fibroblasts in atopic dermatitis

Atopic dermatitis (AD) is a severe autoimmune skin condition characterized by itching and eczematous lesions. It is distinguished largely by epidermal barrier failure and immunological changes, characterized by a predominance of skin-homing Th2 cells that generate IL-4 and IL-13 (41–43).

The degradation of the skin barrier is the direct cause of AD. Kwon indicated that HDAC6 and CXCL13 were increased in AD fibroblasts and enhanced cellular interactions between mast cells, keratinocytes, leading to impaired skin barrier function (44). Research showed that IL-22 increased in the skin and blood of AD patients, and the elevated IL-22 concentrations are highly correlated with skin barrier defects (41). In the skin, IL-22R is primarily expressed by fibroblasts, and its interaction with IL-22 results in increased fibroblast activity, which may lead to skin barrier failure (45). Thus, IL-22 facilitates the communication between leukocytes and fibroblasts, thereby breaking the homeostasis in the skin.

Fibroblasts and their communication with immune cells play an important role in AD. Ghosh et al. discovered that macrophage, endothelial cell, and fibroblast activation pathways, like the NF-κB pathway, played a critical role in AD after reviewing multi-omics research on the disease (46). Another study found that depletion of the *Ikkb* gene in fibroblasts resulted in an atopic dermatitis-like skin phenotype that exhibits eosinophilia and large numbers of type 2 immune cells (14, 47). This effect is related to fibroblasts aberrantly expressing CCL11 to initiate an eosinophilic and Th2 inflammation (14). Fibroblasts-secreted IL-33 also plays an important role in type-2 innate immunity by activating allergic inflammation-related immune cells (48). Furthermore, Fibroblast-generated IL-37b regulates intracellular AMP-activated protein kinase (AMPK) and mammalian target of rapamycin (mTOR) signaling pathways. This regulation can potentially reduce the production of pro-inflammatory cytokines and chemokines associated with atopic dermatitis (AD) by regulating autophagy (49). In AD lesions, various cell types express interleukin-15 (IL-15), including keratinocytes, CD1a^+^ dendritic cells (DCs), CD11b^+^ DCs, CD68^+^ macrophages, and vimentin^+^ fibroblasts (50). In Clark’s study, it was observed that regulatory T cells had an increased proliferation rate when co-cultured with dermal fibroblasts and IL-15, even without any specific antigen stimulation (51). Such findings highlight the importance of cytokines in regulating the immune response in AD and confirmed that targeting cytokines signaling was a promising therapeutic approach for this condition.

Numerous cell groupings in the skin of AD have been revealed by the scRNA-seq. Helen et al. revealed that a novel fibroblast population with COL6A51^+^ and COL18A11^+^ was increased in AD lesions, which expressed inflammatory cytokines induced by type 2 inflammation signal (13). COL6A51^+^ fibroblasts mainly enriched in the upper dermis near the dermal-epidermal junction, and these fibroblasts were adjacent to the CD3^+^ T cells, which suggests a role in recruiting T cell (13). Li’s team found that the expression of interferon-induced genes, like IFITM2 and IFITM3, was enhanced in AD dermal fibroblasts (52). In this study, POSTN was found in fibroblasts from patients with severe AD, which was related to the severity of AD (52, 53). Ko and Merlet uncovered a novel Prx1^+^ fibroblast subpopulation in which the IKKb-NF-κB axis disturbance may affect skin homeostasis and induce an inflammatory condition similar to atopic dermatitis (14). However, the scRNA-seq technique cannot distinguish the cell from the epidermis or dermis, and lack of direct evidence to prove cell–cell interactions that affect proinflammatory and transcriptional networks inside the tissue.

Spatial transcriptomics analysis was used to solve the problems mentioned above. Mitamura et al. have shown that in AD skin, COL6A5, COL4A1, TNC, and CCL19 are increased in COL18A1^+^ fibroblasts in the leukocyte-infiltrated areas through spatial transcriptomics analysis (54). Consistent with previous research, COL18A1^+^ fibroblasts are grown across the whole dermal. However, the activated COL18A1^+^ fibroblasts are particularly localized in the leukocyte-infiltrated area in lesional skin and colocalized with LAMP3^+^ DCs (54). In AD lesions, the interaction between CCL19 produced by the inflammatory COL6A5^+^ COL18A1^+^ fibroblasts subpopulation and CCR7 on T cells and LAMP31 DCs is critical for regulating lymphoid cell organization and trafficking. The type 2 chemokine CCL2 was abundantly expressed by inflammatory fibroblasts, which may regulate macrophages and DC functions. On the contrary, lesional T cells highly expressed the key type 2 cytokine IL13, signaling via IL4R and IL13RA1 on fibroblasts (13).

### 3.3 Dermal fibroblasts in vitiligo

Vitiligo is the most common acquired autoimmune depigmented skin disorder and affects approximately 0.5–2% of the population worldwide (55, 56). Thus far, dermal fibroblasts have been acknowledged for regulating epidermal pigmentation (57) and regulating CD8^+^ T cells in vitiligo (10).

Recently, through the use of scRNA-seq on primary human dermal fibroblasts, Xu has identified a subpopulation of IFNγ-responsive fibroblasts that are uniquely responsible for recruiting and activating CD8^+^ cytotoxic T cells by secreting chemokines such as CXCL9 and CXCL10 (11, 58). Meanwhile, recent evidence suggests that type 2 cytokines such as CCL2 and CCL8 are pivotal in shaping the vitiligo microenvironment (59). An *in vitro* experiment demonstrated that IFN-γ stimulation increased the expression of CCL2 and CCL8 by activating the JAK-STAT pathway in vitiligo fibroblasts (12).

Notably, IFN-γ elicits varying responses in fibroblasts derived from distinct skin regions (11). Yokoi et al. noticed that skin tension in lesional skin was more apparent than in perilesional skin of vitiligo patients (60). In another study, Rani et al. demonstrated an increased expression of collagen type 1 in the lesional skin of vitiligo patients (61). Instead, the expression of collagen type IV, fibronectin, elastin, and adhesion components was considerably lower in the lesional skin of non-segmental vitiligo (NSV) patients (61). Zou et al. found that fibroblasts from vitiligo patients significantly express occludin, suggesting its potential function in the continuous retention of CD8^+^ T cells within the lesions (62). Consequently, the secretion of ECM by fibroblasts exhibits regional variations, potentially contributing to the disparate incidence of vitiligo across different anatomical regions.

Oxidative stress is one of the most essential causes of vitiligo, which leads to the loss of melanocytes and dysfunction of fibroblasts (63). Recent studies have revealed that fibroblasts were associated with the occurrence of oxidative stress in the damage of vitiligo. Yokoi et al. discovered that anti-oxidative action and collagen production were upregulated and collagen degeneration was attenuated in the vitiligo dermis. Furthermore, the expression levels of collagen-related genes and anti-oxidative enzymes were upregulated in vitiligo-derived fibroblasts (60, 61). Despite available data indicating a potential association between fibroblast-produced ECM and vitiligo, further investigation is required.

Indeed, oxidative stress leads to increased metabolic dysregulation and autophagy in melanocytes and fibroblasts. Kovacs discovered that non-lesional vitiligo fibroblasts display increased basal ROS levels associated with the upregulation of the stress-induced marker p53 (64). The authors proposed that autophagy in melanocytes and fibroblasts from non-lesional vitiligo skin is part of a broader metabolic program and may serve as a compensatory/protective response to intrinsic metabolic vulnerability. Inhibition of autophagy exacerbates the dysfunction of vitiligo fibroblasts (65). In addition, Peng has revealed that Lycium barbarum polysaccharide (LBP) protected keratinocytes and fibroblasts against oxidative stress. It was speculated that this protective effect might be attributed to regulating the STAT3-Hsp70-CXCL9/CXCL10 pathway (66).

### 3.4 Dermal fibroblasts in scleroderma

Scleroderma (Systemic sclerosis, SSc) is a chronic multisystem autoimmune disease with high mortality rates (67). At present, dysregulation of fibroblast, increasing ECM, and immune system abnormalities have long been recognized in SSc. Previous studies have demonstrated diverse fibroblasts in SSc exhibited distinct gene expression modules and functions (15).

Cytokines secreted by fibroblasts play an important role in SSc (68). TNF-α stimulation led to an upregulation of MMP-1 secretion by fibroblasts subsequently reversed upon IL-13 treatment (69). A recent study showed that IL-22R expression was enhanced in dermal fibroblasts, and IL-22 treatment enhanced fibroblast responses to TNF-α and promotes a proinflammatory fibroblast phenotype by facilitating TNF-induced keratinocyte activation (70). Meanwhile, type I IFNs enhanced the inflammatory potential of the dermal fibroblast by upregulation of TLR3 and activated its downstream responses (71). Experimental evidence demonstrated that interferon regulatory factor 7 (IRF7), a pivotal regulator of type I interferon signaling, was upregulated in SSc skin, interacted with Smad3, and enhanced TGF-β-mediated fibrosis (72). In animal models of skin fibrosis through TGF-β-dependent pathways, IL-17A has been implicated as a profibrotic mediator that promotes collagen deposition (73). In addition, fibroblasts activated by IL-17 support the growth and differentiation of immune cells (74). Fukasawa has revealed that brodalumab could potentially reduce dermal fibrosis by directly inhibiting the action of IL-17 on fibroblasts (75). Interestingly, in the SSc mouse model, IL-23 from topo I-reactive B cell exacerbated fibrosis (76). In clinical trials, three cases of psoriasis complicated by SSc were treated with guselkumab, an IL-23 inhibitor, and were found to have therapeutic effects on both PsV and SSc (77). Thus, biologics targeting IL-17 or IL-23 may be effective against SSc. Denton showed that endothelial cells-derived cytokines, such as IL-1 and bFGF, modulated fibroblast characteristics. Moreover, lesional scleroderma strains exhibit heightened susceptibility to regulation induced by endothelial cells compared to control fibroblasts (78).

Since long, α-smooth muscle actin (αSMA) myofibroblast is the main type of cells promoted fibrosis in SSc (79). Tabib et al. showed that only a fraction of SFRP2hi SSc fibroblasts differentiate into myofibroblasts, which expressed SFRP4 and FNDC1 (16). Recently, Gur and Wang reported a LGR5^+^ fibroblast subtype which might be the hub of SSc (15). Like other LGR5-expressing mesenchymal cells, the SSc-related LGR5^+^ fibroblast was important for coordinating the correct tissue organization and homeostasis (80). In contrast, Clark reported that MGST1^+^CCN5^+^ fibroblasts had the highest expression of LGR5 in healthy control and later stage of diffuse cutaneous systemic sclerosis (81). Thereby, regulation the function of different fibroblast subsets holds potential as an appealing therapeutic target for systemic sclerosis.

## 4 Conclusion

The last several decades have seen remarkable progress in comprehending the role of fibroblasts in both physiological and pathological conditions within the skin. The field has progressed from phenotypic studies of cultured cells performed more than a century ago to complex genetic and functional observations *in vivo* that have been facilitated by new methods and techniques. These advances have revealed unexpected similarities and unique characteristics of fibroblasts across diverse autoimmune skin diseases, such as psoriasis, AD, vitiligo, and scleroderma, that are currently being leveraged for the treatment of these diseases.

## Author contributions

ZS: Data curation, Visualization, Writing – original draft, Writing – review & editing. ZL: Writing – review & editing. YW: Writing – review & editing. RZ: Writing – review & editing. YD: Writing – review & editing. DL: Conceptualization, Writing – review & editing.
